# Supplementary material for: Enhancing Nurse Practitioners’ Emergency Care Competency and Self-Efficacy Through Experiential Learning: A Single-Group Repeated Measures Study
Source: Healthcare (Basel). 2024 Nov 22;12(23):2333. doi: 10.3390/healthcare12232333 (PMC11641381; doi:10.3390/healthcare12232333)
Supplement: Supplementary file 1 [file healthcare-12-02333-s001.zip › healthcare-3251033-supplementary.pdf]

Emergency Care Competency Checklist

Name : ________________ **Date :** ________________

Item	Not Achieved	Partially Achieved	Fully Achieved
**Emergency Procedure and Operation**			
1. Tap the patient's shoulder and call out to them			
2. Check carotid pulse and assess breathing for 5-10 seconds			
3. Announce the absence of a pulse to the team			
4. Instruct one nurse to begin chest compressions			
5. Instruct one nurse to perform Ambu bagging			
6. Direct two-person resuscitation following the 30:2 ratio			
7. Instruct one nurse to ensure IV patency			
8. Instruct one nurse, or personally connect, the AED			
9. Set up AED functions			
**Problem-Solving**			
10. Move the bed, BVM positioned at the head end			
11. Ensure chest compression position is monitored for accuracy			
12. Instruct insertion of the backboard			
13. Instruct to withhold blood draws initially			
14. Remove the pillow			
15. Correct the mask seal technique			
16. Correct the bagging speed			
17. Instruct two-person Ambu bagging			
18. Correct the chest compression rate			
19. After two minutes, instruct to switch compressors and analyze rhythm			
**AED Operation**			
20. Instruct the team not to touch the patient during AED rhythm analysis			
21. Operate AED and perform defibrillation			
22. Ensure the team steps away before defibrillation			
23. Immediately perform 30:2 chest compressions and ventilation after defibrillation			
**Communication**			
24. Actively inquire about the patient's medical condition from the nurse			
25. Use ISBAR format when handing off to the attending physician			
26. Use closed-loop communication during the resuscitation process			

Evaluator: ____________________
